# Treatment of bone loss in patients with chronic liver disease awaiting liver transplantation

**DOI:** 10.1186/2047-1440-1-7

**Published:** 2012-06-01

**Authors:** Daniel Kaemmerer, Benjamin Schmidt, Gabriele Lehmann, Gunter Wolf, Utz Settmacher, Merten Hommann

**Affiliations:** 1 Department of General and Visceral Surgery, Zentralklinik Bad Berka GmbH, Robert-Koch-Allee 9, Bad Berka, 99437, Germany; 2 Department of Internal Medicine III, University Hospital Jena, Jena, Germany; 3 Department of General, Visceral and Vascular Surgery, University Hospital Jena, Jena, 07747, Germany

**Keywords:** Bone mineral density, Osteoporosis, Ibandronate, Liver transplantation, Chronic liver disease

## Abstract

**Background:**

Most of the patients awaiting liver transplantation already have osteopenia or even osteoporosis by end-stage liver disease.

In a retrospective study, we investigated the effect of pre-treatment with oral monthly ibandronate (150 mg), vitamin D3 (800 IU/day) and calcium (1 g/day) for osteopenia and osteoporosis caused by end-stage liver disease in patients before and after liver transplantation (LT).

**Methods:**

The bone mineral density (BMD) of the lumbar spine (LS) and the femoral neck was measured prospectively pre- and post-LT in 31 patients with existing pre-transplant osteopenia. Patients had osteopenia of the LS prior to LT (T-score −1.8 ± 1.5) so that the treatment medication was initiated immediately after the diagnosis.

**Results:**

The study group showed a permanently increased BMD with significant differences (g/cm²) from baseline up to 12 months post LT at the lumbar spine (LS: pre-LT 0.80 ± 0.11 g/cm², three months: 0.90 ± 0.08 (*P* <0.005); six months: 0.95 ± 0.11 (*P* < 0.008); 12 months: 1.00 ± 0.09 -0.85 (*P* <0.012).

**Conclusion:**

The combined pre- and post-operative treatment with oral ibandronate had significantly improved bone mineral density of the lumbar spine at 3, 6 and 12 months post LT. The immediate post-operative bone loss after LT can be significantly avoided by pre-treatment of liver transplant candidates affected by osteopenia.

## Background

Numerous patients have osteoporosis before transplantation as a result of their underlying chronic hepatic disease. The reported numbers range from 12% up to a considerable 55%, depending on the study [1-4]. With respect to the frequency of bone fractures in chronic liver disease, the reports range from 3% to 35% [5-7]. The type of osteoporosis in chronic liver disease cannot be determined clearly, as aspects of ‘low turnover’ osteoporosis as well as characteristics of ‘high turnover’ osteoporosis have been reported [5,8]. It has been demonstrated that the bone status before transplantation is a predictive factor for the bone mineral loss after liver transplantation (LT) [9]. Low bone mineral density (BMD) values at the time of evaluation for transplantation or bone fractures prior to transplantation increase the risk of suffering further BMD loss after LT. The first three to six months after transplantation are associated with the greatest loss of BMD [10-13]. The probability of bone fractures in the course of the post-transplant period has been reported as ranging from 6% to 65% [14-17]. Studies which initiate treatment at the time of osteopenia diagnosis prior to transplantation are missing to date.

We present our results of a retrospective study for the evaluation of an oral monthly dose of ibandronate as an immediate bisphosphonate treatment of pre-existing osteopenia in patients awaiting LT who were followed-up for up to 24 months after LT.

## Patients and methods

Patients with chronic liver disease who underwent LT between May 2006 and December 2008 at the University Hospital Jena were enrolled. Informed consent was obtained of every patient who was evaluated for LT. Ethical approval was given by the local committee. All patients underwent a BMD measurement of the lumbar spine (LS L1-L4) and of the femoral neck (FN) with dual-X-ray-absorptiometry (DEXA, Lunar Prodigy Advance/Narrow Fan, General Electric, USA) during their evaluation for LT and post-operatively at three, six, 12 and 24 months. Laboratory tests of bone metabolism parameters (bone-specific alkaline phosphatase (BAP), pyridinoline (PYD), and deoxypyridinoline (DPYD)) were performed at the same points in time. The study group consisted of 31 patients (age: 54.1 ± 9.5 years, Table 1) who had osteopenia at the LS at the time of evaluation (a densiometric T-score lower −1.5).

**Table 1:** Patients’ characteristics before transplantation

	**IBA**
**Age (years)**	54.1±9.5
**Total (n)**	31
**Female/male**	9 / 22
**Liver disease**	cholestatic: 2 non-cholestatic: 29
**T-score lumbar spine** **( 0 – -1 normal range)**	-1.77±1.51
**T-score femoral neck**	-1.21±1.24
**Bone specific alkaline phosphatase (8-16.6 μg/l)**	18.61±8.74
**Pyridinoline** **(95 – 215 μg/g creatinine)**	311±180
**Desoxypridinoline (15 – 45 μg/g creatinine)**	46.01±28.2

IBA, ibandronate.

T-score is a comparison of a patient’s BMD to a healthy, young reference group with the same sex and ethnicity. Osteopenia is defined as a BMD T-score between −1.0 and −2.5 and osteoporosis as a T-score below −2.5 standard deviations below the mean peak bone mass. In these patients, the treatment (oral monthly IBA 150 mg, vitamin D3 800 I**E** and calcium 1000 mg daily) was started before LT (an average of 18 months before LT). Immunosuppression after LT was managed as quadruple induction with steroid, tacrolimus, mycophenolate mofetil and basiliximab. Afterwards, treatment was individualized. Except in patients with an underlying auto-immune disease, steroid withdrawal was attempted in all patients within the first six months post-LT.

## Laboratory tests

At the time of evaluation for LT as well as three, six, 12 and 24 months after transplantation, the following laboratory parameters were measured: serum bone-specific alkaline phosphatase (8 to 16.6 μg/l), early morning urinary excretion PYD (95 to 215 μg/g creatinine), and DPYD (15 to 45 μg/g creatinine).

## Fracture analysis

X-ray and computed tomography checks were routinely performed before LT to rule out pre-existing fractures. In the follow-up period, X-ray and computed tomography were performed in cases of clinical symptoms with regard to bone fractures or in cases of bone densiometric suspicion of a bone fracture. Fracture information was taken from patient records as well as by phone interviews after 24 months post-LT.

## Statistical analyses

Primary endpoints were changes in bone mineral density of the LS at 3, 6, 12 and 24 months post-LT. Occurrence of bone fractures and changes in the bone mineral density of the FN were considered as secondary endpoints.

Primary endpoints were changes in BMD of the LS at three, six, 12 and 24 months post-LT. Occurrence of bone fractures and changes in the bone mineral density of the FN were considered as secondary endpoints.All parameters were initially checked with the Kolmogorov-Smirnov adjustment test for normal distribution. The BMD differences and the laboratory parameters were analyzed by paired or un-paired t-test, Mann–Whitney test or Wilcoxon test depending on the status of the normal distribution.

## Results

The patient characteristics of the groups are presented in Table 1.

### Bone mineral density measurement

The BMD measurements are presented in Table 2.

**Table 2:** Bone mineral density of the lumbar spine and femoral neck

		**T-scores**	***P*-value T-score vs. Pre-LT**	**g/cm²**	***P*-value g/cm² vs. Pre-LT**	**Percentage changes from baseline**
**Spine L1-L4**	Pre-TX	-1.77±1.51	-	0.80±0.11	-	-
	3 months	-1.31±1.52	0.001	0.90±0.08	0.005	13.59±12.32
	6 months	-1.02±1.53	0.005	0.95±0.11	0.008	17.10±12.68
	12 months	-0.85±1.18	0.005	1.00±0.09	0.012	18.78±14.98
	24 months	-0.87±1.11	0.08	1.03±0.10	0.068	24.26±15.61
**Femoral neck**	Pre-TX	-1.21±1.24	-	0.74±0.14	-	-
	3 months	-1.22±1.19	0.001	0.75±0.10	0.929	3.11±9.30
	6 months	-1.32±1.09	0.001	0.83±0.13	0.443	5.08±10.06
	12 months	-1.24±0.80	0.001	0.85±0.10	0.450	3.31±10.05
	24 months	-1.30±0.64	0.002	0.93±0.09	0.783	2.72±12.83

LT, liver transplantion; TX, transplantation.

At the LS the study group showed a consistently significant increase in BMD from three to 12 months (T-scores and g/cm²). With regard to baseline, we observed a significant BMD rise of T-scores from three to 24 months. The g/cm² data demonstrated a persistent rise in BMD but without significant differences at FN.

### Bone fractures

Within 24 months follow-up after transplantation, there was one osteoporosis-associated pathological bone fracture (1/31; 3.2%) without corresponding trauma. There was one vertebral body fracture and no extravertebral fractures.

### Laboratory test results

Bone specific alkaline phosphatase, Pyridinoline, and Deoxypyridinoline

The laboratory chemistry parameters are shown in Table 3.

**Table 3:** Pyridinoline (PYD μg/g creatinine) and Deoxypyridinoline (DPYD μg/g creatinine), Bone specific alkaline phosphatase (BAP μg/l).

**PYD** **(μg/g creatinine)**	**IBA**	***P*-value vs. Pre-LT**	**DPYD** **(μg/g creatinine)**	**IBA**	***P*-value vs. Pre-LT**
**Pre-LT**	311±180	-	**Pre-L**	46.1±28.2	-
**3 months**	301±118	0.51	**3 months**	35.4±14.0	0.44
**6 months**	317±146	0.31	**6 months**	42.8±19.5	0.49
**12 months**	220±62	0.18	**12 months**	33.4±17.4	0.75
**24 months**	169±54	0.18	**24 months**	32.2±15.6	0.65
**BAP** **(μg/l)**	**IBA**	**p-value ** **vs. Pre-LT**			
**Pre-LT**	18.6±8.7	-			
**3 months**	10.0±3.9	0.016*			
**6 months**	12.2±3.9	0.022*			
**12 months**	14.9±5.8	0.08			
**24 months**	15.0±7.7	0.63			

IBA, ibandromate; LT, liver transplantation.

The study group noted elevated levels of PYD, DPYD and BAP before transplantation. PYD values were above the normal range and slowly declined up to 12 months post-LT whereas BAP and DPYD showed normal values directly after LT.

## Discussion

In this study, we have evaluated the effect of an oral bisphosphonate ibandronate, vitamin D3 and calcium treatment for the treatment of preexisting osteopenia and osteoporosis in patients awaiting liver transplantation. As far as we are aware, this is the first study to clinically evaluate the use of pre-treatment with ibandronate before LT. Osteopenia and osteoporosis in patients with end-stage liver disease is a well known clinical problem, frequently associated with cholestatic diseases but also found in patients with hepatitis C, alcoholic liver disease and even hemochromatosis as reasons for LT [2,18].

We have demonstrated that monthly ibandronate treatment (150 mg orally) in combination with calcium (1000 mg) and vitamin D3 (800 IU) is a promising treatment approach for the reduction of post-transplant osteopenia and osteoporosis after LT. At none of the measurement points in time post-operatively, were there BMD losses in the LS and FN.

In the pre-treated group, we saw marked BMD increases, in particular at the LS but also at the FN; however, BMD increased to a lesser extent in the FN. It was possible for the first time, to modulate the immediate post-operative BMD loss in favor of a marked BMD increase (LS: three months: 13.6%, six months: 17.1%; FN: three months: 3.1%, six months: 5.1%) by initial ibandronate treatment at the time of the evaluation for liver transplantation.

### Bone mineral density measurements

Different studies have been published on the treatment of BMD loss in patients with chronic liver diseases. Intravenous as well as oral bisphosphonates have been proven to be effective in chronic liver diseases and after LT but with marked qualitative differences. Most of the studies focus on patients with primary biliary cirrhosis (PBC) as one entity for chronic liver diseases which could be treated by LT [5,8]. PBC-associated bone loss can be significantly improved by alendronate compared to placebo, as shown by Zein *et al*. [19]. In a study by Guanabens *et al*. alendronate was proven to be more effective in reducing bone loss than treatment with etidronate [20]. The greater antiresorptive power has been shown to be one reason for the higher efficacy of alendronate.

Our study confirms the efficacy of ibandronate for increasing BMD at the LS, as has been reported by several previous studies [21-23]. Ibandronate and zoledronate are bisphosphonates with the highest antiresorptive potential which could result in such high bone increases as reported by our data and the data of Bodingbauer *et al*. up to 24 months after LT [24]. Many studies have shown that the BMD loss is highest during the first three to six months after transplantation. This is particularly evident at the spine. It correlates also with the incidence of vertebral body fractures shortly after LT [17,25]. It shows the need for sufficient bone loss protection during the first months after LT. In contrast to a review by Kasturi *et al*. [26] who found bisphosphonate use did not result in a statistically significant change in BMD at the end of the first year after LT, our study shows a significantly permanent increase up to 12 months post-LT at the LS for the T-scores as well as the percentage measurements (g/cm²).

Pre-treatment with ibandronate has shown an excellent and highly significant BMD increase at the LS and the FN, particularly during the critical immediate post-operative period. Post-operative ibandronate treatment was proven to be effective and BMD increases were recorded. These results were confirmed by Wagner D *et al*. who observed a marked increase in BMD and a significantly lower rate of fractures after ibandronate treatment [27].

In a randomized controlled prospective trial, Bodingbauer *et al*. showed the effectiveness of a prophylactic bisphosphonate treatment with zolendronate during the first 12 months after LT [24]. They not only reported on high elevated BMDs, they also proved the potency of fracture reduction. This study underlines our data with ibandronate. We have shown highly elevated bone mineral density at the LS and FN and only reported one fracture during the course of the investigation. In our study, the prevalence of bone fractures was 3.2%. This result seems to confirm the efficacy of ibandronate for the reduction of bone fractures after LT as does the prevalence of bone fractures in 7.4% of the patients in the study of our group before ibandronate intravenously 2.0 mg every three months [28]. It has to be recognized, however, that this study is retrospective, underpowered and not designed for proving a decrease of fractures post-LT. It became evident that the bone status before LT is an important predictive factor for the loss of BMD after LT. Low BMD values at the time of evaluation for LT or previous bone fractures increase the risk of suffering further BMD loss after LT [9]. The early treatment of osteopenia and osteoporosis increases the BMD and avoids the decline caused by immobilization, high steroid and calcineurin-inhibitory medication during the first months post-LT. The question about the limitation of post transplant fractures has to be proven in further studies with an adequate number of cases.

### Laboratory tests

The study group showed an increased BAP activity prior to treatment initiation with ibandronate/vitamin D/calcium. The vitamin D status at the time of evaluation for LT was unknown and this is a limitation of the study. As it is known that a vitamin D deficiency is frequently observed in patients with chronic liver failure [29], this may be assumed with a high probability as being the cause of the increased BAP activity. There was a reduction of the BAP activity in due course in the study group, even though this was less pronounced. This may reflect a reduced bone turnover under the influence of ibandronate and it may be the result of the mineralization promoting effect of vitamin D treatment. As for DPYD, there is no certain anti-resorptive effect on the bone turnover evident, contrary to our expectations. These findings are in agreement with the results of an investigation by Bodingbauer *et al*. who did not find any dynamic of the investigated markers of bone turnover, except for a reduction of osteocalcine [24].

## Conclusions

The combined pre- and postoperative treatment with oral ibandronate significantly improved bone mineral density of the LS at at 3,6 and 12 months post-LT. The immediate post-operative bone loss after LT can be significantly avoided by pre-treatment of transplant candidates affected by osteopenia.

## Competing interests

One author of this manuscript has conflicts of interest to disclose.

Dr. Kaemmerer has been supported by travel funds provided by Hofmann-La Roche Ltd. and Bayer AG.

All other authors declare that they have no competing interests.

## Authors’ contribution

DK, GL and MH designed the study. DK and BS performed the study and wrote the manuscript. Transplantations were arranged by US, densiometric measurements held by GL and GW. All authors read and approved the final manuscript.
